# Aspirin Resistance

**DOI:** 10.1155/2009/937352

**Published:** 2009-04-14

**Authors:** Khaled Mansour, Ali T. Taher, Khaled M. Musallam, Samir Alam

**Affiliations:** ^1^Division of Cardiology, Department of Internal Medicine, American University of Beirut Medical Center, Beirut 1107 2020, Lebanon; ^2^Division of Hematology-Oncology, Department of Internal Medicine, American University of Beirut Medical Center, Beirut 1107 2020, Lebanon

## Abstract

The development of adverse cardiovascular events despite aspirin use has established an interest in a possible resistance to the drug. Several definitions have been set and various laboratory testing modalities are available. This has led to a wide range of prevalence reports in different clinical entities. The etiologic mechanism has been related to clinical, genetic, and other miscellaneous factors. The clinical implications of this phenomenon are significant and warrant concern. Management strategies are currently limited to dosing alteration and introduction of other anitplatelet agents. However, these measures have not met the expected efficacy or safety.

## 1. Introduction

Despite the development of newer antiplatelet drugs in the last decade, aspirin is still the most widely used antiplatelet agent across the world to prevent cardiovascular diseases [1–4]. In the 19th century, willow leaves became an attraction after their extract, salicylic acid, was found effective as an analgesic for arthralgias and an antirheumatic for a variety of rheumatic disease [5–7]. At the end of the 19th century the acetylated form of salicylic acid was manufactured [8], with less gastrointestinal side effects, and consequently became more widespread and commonly used. Long-term aspirin administration in patients at high risk of occlusive vascular events reduced up to 34% of nonfatal myocardial infarction (MI), 25% of nonfatal stroke, and 18% of all-cause mortality [4]. Ever since, several patients have reported developing adverse vascular events despite aspirin intake, an observation that was later coined the term “aspirin resistance” (AR) [9]. Nowadays, the term has been employed to express the occurrence of cardiovascular events in spite of regular intake of aspirin at recommended doses [10–13]. Recent advances in evaluating platelet function and the introduction of the point-of-care platelet function machinery made assessing the degree of platelet response to a certain antiplatelet drug more reasonable, accessible, and easier to perform [14].

In this paper, the prevalence, mechanism, and clinical implications of aspirin resistance will be highlighted. Moreover, the available laboratory tests used to assess this phenomenon and the possible ways to overcome it will be described.

## 2. Terminology

The lack of agreement on a standardized definition for “aspirin resistance” has contributed to the disparity in reports of its incidence among different studies. Whereas some use the term “aspirin treatment failure,” while others like to call it “aspirin nonresponsiveness.” The term “resistance” was assigned based on biochemical and laboratory findings in which aspirin was unable to inhibit one of the many available in vitro tests of platelet function [15, 16]. Hence, from a pharmacologic point of view, resistance to aspirin may be defined as lack of ability to attain the expected inhibition of platelet cyclooxygenase-(COX-)1 with avoidance of platelet thromboxane (TX) A2 formation [17]. “Aspirin treatment failure” is defined based on clinical outcomes, when aspirin fails to prevent recurrent vascular ischemic events. However, reinfarction after aspirin use in the setting of an acute coronary event may be due to thrombus spread mediated by adenosine diphosphate (ADP) rather than continuing TX-induced platelet aggregation; which may render the terminology “failure” improper [18]. Some suggested that until the various possible reasons of treatment failure with aspirin have been recognized, the more suitable term may be “aspirin nonresponsiveness” [15].

## 3. How Prevalent Is Aspirin Resistance?

Due to the lack of standardized testing for AR, prevalence rates of nonresponders to aspirin among adults differ according to the platelet function test used and the threshold of response, with a wide range reported (5.5 to 60%) [19–22]. For instance, there are up to seven different thresholds for defining aspirin response using the PFA-100 [23–29]. When using the combination of different laboratory tests to define resistance (VerifyNow-Aspirin, optical aggregometry, and PFA-100), a lower resistance rate (2%) was reported as compared to using each test alone [30].

The majority of studies on AR were conducted on adult patients, but recently the prevalence of AR was studied in 44 children aged 1 to 17 years taking aspirin for different indications, by using different laboratory tests. Six out of 44 were considered aspirin resistant according to at least one laboratory test (5 by PFA-100, 1 by aggregometry, and urinary 11dhTxB2), which leads to the conclusion that, as with adults, the incidence of AR is also assay-dependent in the pediatric population [31]. Table 1 summarizes the main studies investigating AR prevalence in different clinical entities [28, 30, 32–36].

## 4. Proposed Etiology

### 4.1. Pharmacology of Aspirin

Low-dose aspirin (as low as 81 mg) irreversibly inhibits the COX-1 enzyme, by acetylating the serine residue at position 529, consequently impairing the transformation of arachidonic acid to prostaglandin (G2/H2), and TX A2, which is a potent mediator of platelet aggregation and activation. This effect explains the clinical benefit of aspirin in patients with high risk vascular disease [37–39]. Aspirin's effect on COX-2 is minimal in doses <1200 mg [40, 41].

### 4.2. Mechanism of Resistance

The different mechanisms by which AR might take place are not yet well understood [10, 12, 13, 19]. Medication compliance is one preventable and important contributor to the phenomenon of resistance and to the overreporting of aspirin nonresponsiveness [42]. To stress the importance of this factor, a recent meta-analysis including 50 000 patients at high risk of ischemic coronary disease found that noncompliance or early discontinuation of the drug carried a 3 times higher risk of cardiac events (odds ratio [OR] 3.14, 95% confidence interval [CI] 1.75–5.61; *P* = .0001) [43]. Thus, explaining the benefits of antiplatelet therapy to the patient may help improving compliance [44].

Some drugs may compete with aspirin at the COX-1 receptor site; of those, the most commonly encountered are nonsteroidal anti-inflammatory drugs such as ibuprofen which can offset the clinical benefit of aspirin in a variety of vascular diseases [45]. The bioavailability of a drug is dependent on numerous factors, absorption being the most relevant. Lower doses of acetylsalicylic acid may be hydrolyzed to a higher extent into an inactive form, by gastrointestinal mucosal esterases when given with proton pump inhibitors due to acid suppression, thus reducing the absorption of the active drug. However, current evidence failed to confirm this argument [46, 47].

Another hypothesis considers resistance at the target site of the drug's action. This may highlight the role of genetic studies to determine the potential contribution of some genetic polymorphisms in AR. Polymorphisms in the COX-1 gene have been implicated in the partial nonresponse to low-dose aspirin [48, 49]. However, a recent large systematic review addressing the role of different genetic polymorphisms did not find a clear association between COX-1 gene polymorphisms (specifically C50T/A842G polymorphism) and AR [50]. Another major genetic contributor to biological AR is thought to be the PlA1/A2 polymorphism in the GPIIIa platelet receptor [51–53], which is according to the same systematic review, the most frequently investigated parameter (19 studies involving 1389 subjects) [50]. This variant was noticeably associated with AR when measured in the healthy population (OR 2.36, 95% CI 1.24–4.49; *P* = .009), but combining data from both healthy individuals and those with cardiovascular disease reduced the size of the observed effect (OR 1.14, 95% CI 0.84–1.54; *P* = .40) [50].

Poor glucose control and body weight are also proposed to contribute to AR, where in a recent study assessing 48 patients with type 2 diabetes mellitus using the PFA-100 assay, AR was significantly associated with HbA1c ≥8% (*P* = .002) and obesity (BMI ≥ 30 kg/m^2^; *P* = .01) [54]. Although this might implicate that better glucose control leads to less incidence of AR, the clinical significance of such findings should be carefully inspected, since in two of the largest trials [55, 56] assessing the role of aspirin on primary prevention of cardiovascular events in patients with type2 diabetes, low-dose aspirin did not decrease the risk of cardiovascular events when compared to placebo (13.6 per 1000 person-year in the aspirin group versus 17.0 per 1000 person-year in the placebo group, hazard ratio [HR] 0.80; 95% CI 0.58–1.10 in the JPAD trial [55]; 116 of 638 primary events in the aspirin group compared with 117 of 638 in the no aspirin group, HR 0.98; 95% CI 0.76–1.26 in the POPADAD trial [56]). In the JPAD trial both groups (aspirin versus no aspirin) had similar baseline characteristics in terms of glycosylated hemoglobin (7.0 versus 7.1, resp.). On the other hand, the aspirin group in the POPADAD trial had a mean HbA1c of 8.0 compared to 7.9 in the no aspirin group. The lack of beneficial effect of aspirin in the latter study may be partly explained by the fact that AR is significantly associated with HbA1c levels ≥8% [54].

**Figure 1:**
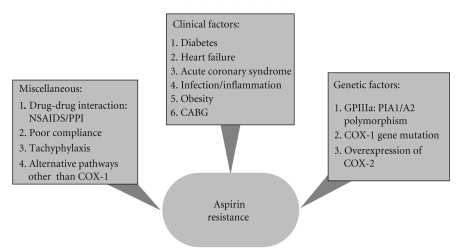
Proposed factors contributing to aspirin resistance (CABG: coronary artery bypass grafting; COX: cyclooxygenase; NSAIDS: nonsteroidal anti-inflamatory drugs; PPI: proton pump inhibitors).

Finally in all conditions associated with a high platelet turnover (coronary artery bypass grafting, acute coronary syndrome (ACS), acute or chronic infection, or inflammation), low-dose aspirin is associated with a short half-life (15 to 20 minutes) and might not be able to suppress COX-1 in fresh platelets that are continuously and quickly released into the circulation in such stressful circumstances, leading to higher platelet reactivity (Figure 1) [57–60].

## 5. Platelet-Function Testing

Multiple assays for platelet function and response to aspirin have emerged in the past decade. The test that is considered gold standard for assessment of the degree of aspirin response is light transmittance aggregometry (LTA) [61]. This assay measures the increase in light transmittance across platelet-rich plasma as a consequence of aggregation of platelets and development of clumps in response to different agonists (ADP, collagen, arachidonic acid). Several drawbacks limit the use of this assay. However, it has been described as time consuming, operator dependent, and of high cost. Inability to reproduce results, even in the same laboratory, has also been reported [62]. According to this assay, the most accepted definition of AR is ≥20% platelet aggregation with 1 mg/mL arachidonic acid and ≥70% aggregation with 10 *μ*mol/l ADP despite regular intake of aspirin [63].

The point-of-care platelet function analyzer PFA-100 device (Dade Behring, Leiderbach, Germany) acts like an injured artery, where high shear stress conditions are present, and works in the presence of erythrocytes; unlike LTA where there is no interaction between platelet and other blood components. Hence, platelet function is evaluated by the time needed to form a platelet plug to occlude the gap present in this device. Using this test, AR is generally defined as a closure time of <164 s despite regular aspirin intake. The device is easy to use and requires only a small amount of blood. The test is also fast and reproducible. Unfortunately, correlation between clinical outcomes with the PFA-100 is poor [64]. The lack of correlation with clinical outcome was also demonstrated in a recent prospective study assessing the prevalence of resistance in 97 patients with stable coronary artery disease on 160 mg aspirin for at least one month using the PFA-100 and a follow-up of 2.5 years for the composite of death, MI, and ischemic cerebral infarction or acute limb ischemia. It was found that aspirin resistant patients (29.9%) did not have a higher risk of death, MI, or ischemic vascular event compared with aspirin-sensitive patients [65]. Moreover, the reliance upon hematocrit and plasma von Willebrand factor, along with high cost, limits the use of PFA-100.

Another point-of-care newly introduced assay is the VerifyNow-Aspirin (the Ultegra Rapid Platelet Function Assay, Accumetrics Inc., San Diego, Calif, USA) which correlates well with light transmittance aggregometry [66]. Results from the VerifyNow-Aspirin were highly reproducible in 21 healthy volunteers and 40 patients with stable coronary artery disease [67]. It also showed poor sensitivity and good specificity with a cut-off value at 550 aspirin reaction units (ARU), compared to LTA, which makes the significance of the cut-off level at 550 ARU for detecting AR controversial [67]. 

Other test measures consider the end products of the TX A2 pathway such as serum TX B2 [68], or urine 11-dehydroTX B2 [49], for assessing aspirin activity [49]. In fact, these two tests may better reflect the amount of TX A2 derived from sources other than platelets such as macrophages and monocytes, and on the COX-2 linked pathway of arachidonic acid, which is blocked by aspirin at very high doses (1200 mg) only [40, 41]. Urinary 11-dehydroTX B2 concentration is affected by kidneys production of this substance; however, measurement of this metabolite is still commonly used in trials assessing AR due to its low cost and ease to carry out [49, 69].

**Figure 2:**
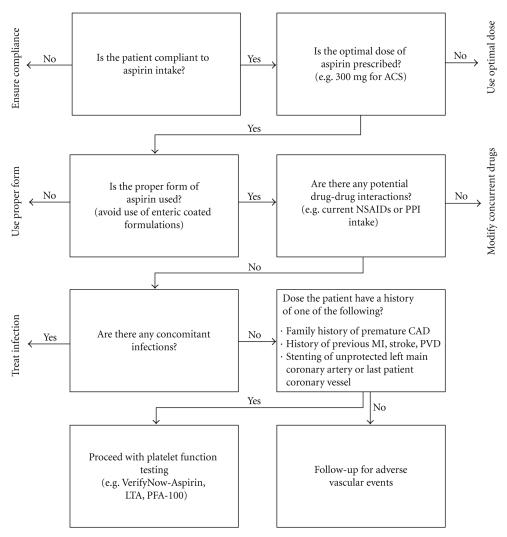
Algorithm highlighting approach to a patient with suspected aspirin resistance. ACS: acute coronary syndrome; NSAIDs: nonsteroidal antiinflamatory drugs; PPI: proton pump inhibitors; CAD: coronary artery disease; MI: myocardial infarction; PVD: peripheral vascular disease; LTA: light transmittance aggregometry; PFA: platelet function analyzer.

A relevant question is the extent to which these laboratory methods correlate with one another. The various laboratory assays used to identify AR are compared weakly with each other. This was demonstrated in a study using six different platelet function tests in 201 patients with stable coronary artery disease who were on daily aspirin use. The encounter of AR varied according to the assay used, being uppermost for the PFA-100 (60%) and lowermost using LTA (4%) [70]. Workup of a patient with suspected aspirin resistance, eventually leading to appropriate platelet-function testing, is highlighted in Figure 2.

## 6. Clinical Implication

Another significant question to consider is whether this phenomenon is confined to laboratory findings or affects the expected clinical outcome. A recent meta-analysis on 2930 patients with cardiovascular disease, who were on aspirin (75–325 mg daily) alone or in combination with other antiplatelet therapy, found resistance to be more prevalent in females as well as in patients with renal impairment. These populations were found to carry a fourfold higher risk of death due to vascular events and higher risk of nonfatal cerebrovascular and cardiovascular events compared to aspirin sensitive patients (39% of AR patients versus 16% of aspirin sensitive patients had a cardiovascular event, OR 3.85, 95% CI 3.08–4.80; *P* < .001), regardless of the assay used to assess resistance [71]. The authors conclusions are reinforced by a previous meta-analysis evaluating clinical outcome in AR, where again, resistant patients had considerably higher risk of recurrent vascular events compared to aspirin sensitive patients [72].

In a recent trial evaluating the relationship between AR (assessed by thrombelastography) and stroke in 45 patients with ischemic stroke, it was found that AR was more frequent in the stroke than the control arm (67% versus 40%; *P* = .028). Within the stroke group, the AR arm had more severe stroke (assessed by Rankin score). In addition AR was greater in lacunar than embolic strokes [73]. In a follow-up of 468 patients with stable coronary artery disease and/or a high risk for vascular events (diabetic and/or hypertensive) for a mean period of 379 ± 200 days, cardiovascular death and/or nonfatal events were more frequent among patients with AR [74]. Moreover, after measuring urinary 11-dehydroTX B2 levels in 976 high-risk patients (half of them had sustained previous vascular events) who were initially enrolled in the heart outcomes prevention evaluation (HOPE) trial (on aspirin for 5 years), it was demonstrated that patients with levels in the highest quartile sustained more MI and cardiovascular death versus those in the lowest quartile [49], the drawbacks of this nested case control study were the issue of compliance which was not verified adequately by objective laboratory methods.

The clinical correlation of AR was also elucidated in patients undergoing percutaneous coronary intervention (PCI) in a recent study that measured the cardiac biomarkers (CK-MB and Troponin I) in 151 patients who underwent nonurgent PCI and who were already on various doses of aspirin and a 300 mg loading dose of clopidogrel. The study demonstrated that patients with AR, assessed using the VerifyNow-Aspirin test, sustained a considerably higher incidence of post-PCI elevation in cardiac biomarkers [75]. Furthermore, in 216 patients with ST elevation myocardial infarction (STEMI), enhanced platelet activity assessed with PFA-100, was an independent predictor of markers of cardiac necrosis [76]. The laboratory response to glycoprotein (GP) IIB/IIIA inhibitors and the clinical outcome in 70 patients with STEMI undergoing PCI correlated well with baseline platelet reactivity (PR) (assessed with the PFA-100) before intervention. High-baseline PR was associated with a 5–11 times increase in the risk of death, reinfarction, and target vessel revascularization (HR 11, 95% CI 1.5–78; *P* = .02 when the PFA-100 was used and HR 5.2, 95% CI 1.1–23; *P* = .03 using the LTA) [77].

Finally, aspirin resistance has been implicated recently as a potential cause of the rare but very serious complication of drug eluting stents thrombosis. This was demonstrated in a prospective systematic analysis of platelet aggregation in four subsequent cases of late thrombosis, where all four cases showed resistance to either aspirin (evaluated with the PFA-100) or clopidogrel, and two cases showed dual resistance to both of these platelet antiaggregants [78].

## 7. Management

After correlating AR with incidence of more adverse cardiovascular events many investigators are trying to find solutions to overcome this poor response. The idea of increasing aspirin dose in order to overcome resistance has been assayed in many studies, since there is some evidence that response to aspirin may be dose-dependent [27]. Laboratory and genetic inconsistency as well as dose dependence is seen when agonists other than arachidonic acid (the most specific in assessing AR), such as (ADP, collagen, epinephrine), are used for in vitro assessment of platelet inhibition by aspirin. This inconsistency and dose-dependence may be explained by the fact that those agonists maybe only in part reliant upon, or even independent of, the COX-1 pathway which is the main target of aspirin [79–82]. More solid evidence that reflects on the appropriate dosage of aspirin was portrayed by the antithrombotic trialists collaboration. It showed that the most effective aspirin dose with the fewest adverse consequences is 75 to 150 mg once daily with similar efficacy as doses up to 1500 mg daily [8, 83]. Even though low-aspirin doses might relate to resistance by reducing absorption, administration of higher doses looks unwarranted and is outweighed by a higher risk of gastrointestinal bleeding [84].

In a number of studies, sensitivity of platelets to ADP and levels of this agonist in patients with AR revealed to be considerably amplified [25, 85]. Moreover, in a randomized cross-over trial, patients with AR turned out to be highly responsive to platelet ADP receptor antagonist [86]. One could argue, based on the above that replacing or adding a different antiplatelet could cancel out the incidence of adverse events resulting from resistance to aspirin. This theory was refuted by a recent meta-analysis of 20 studies (6 of them had an additional antiplatelet used) which found that concomitant treatment with additional antiplatelet (namely, clopidogrel or tirofiban) provided no clinical benefit to those patients identified as aspirin resistant (OR 2.52, 95% CI 1.79–3.56 for aspirin alone versus OR 3.06, 95% CI 1.99 to 4.70 for the dual antiplatelet group) [71]. To elaborate on the issue of dual antiplatelet therapy, an analysis of asymptomatic low-risk patients on both aspirin and clopidogrel in the CHARISMA trial revealed a higher incidence of death due to cardiovascular disease [87], and that dual antiplatelet therapy should be reserved for patients with high risk for vascular events [88]. The addition of dipyridamole to aspirin has also been subject to much debate [4, 89, 90]. In a recent review involving 23 019 patients with vascular disease [91], dipyridamole alone or in combination with aspirin did not lessen the risk of vascular death (relative risk [RR] 0.99, 95% CI 0.87–1.12), though the risk of vascular events was lowered (RR 0.88, 95% CI 0.8–0.95).

In particular, dealing with some patients who develop adverse vascular events (e.g., cerebrovascular accidents (CVA) and ACS) despite being on aspirin remains challenging, and many questions persist without clear cut answers. There is no clear evidence and no consensus yet on whether using different laboratory assays to detect aspirin resistance would guide therapy in those patients, especially after a recent report assessing AR status with PFA-100 in 129 patients with transient ischemic attack (TIA), stroke, or vascular cognitive impairment failed to predict new thrombotic events in patients found to be AR during mean follow-up of 56 months, as new thrombotic events occurred in 15.4% of AR patients and in 14.6% of those without resistance (*P* = 1.00) [92].

Although the subgroup analysis of the CAPRIE trial failed to show a significant beneficial effect of clopidogrel over aspirin in patients with history of recent stroke or MI [93], the recommendations issued by the American Heart Association/American Stroke Association (AHA/ASA) in 2006 on stroke prevention [94] supported initial antiplatelet therapy with the combination of aspirin and extended-release dipyridamole (ER-DP), 25 mg/200 mg twice a day (aggrenox), rather than aspirin (Grade 2A evidence) or the use of clopidogrel for those not treated with aggrenox (Grade 2B evidence) in patients with a history of noncardioembolic stroke or TIA of atherothrombotic, lacunar (small vessel occlusive type), or cryptogenic type. Hence, adding an additional antiplatelet such as (ER-DP) or substituting aspirin by another potent antiplatelet (namely, clopidogrel) would be a logical alternative to patients who develop CVA despite using aspirin on a daily basis. The addition of clopidogrel to aspirin for secondary stroke prevention has fallen out of favor since the combination of the two drugs does not offer better benefit for stroke prevention than either drug alone but does considerably amplify the risk of bleeding complications [95]. Unlike patients who develop stroke, those being on aspirin and develop ACS (unstable angina and non-STEMI) would benefit from adding clopidogrel to aspirin as per the famous CURE trial [96].

## 8. Conclusion

Aspirin resistance is a true phenomenon that needs to be further elucidated. A single definition should be provided when describing resistance or nonresponse to aspirin. Moreover, consensus should be made about the optimal laboratory method that allows objective assessment of response to aspirin. These points, if achieved, may normalize the wide range of prevalence reported among different studies. The correlation between AR and higher incidence of adverse vascular events is established by a considerable number of well-designed trials. On the other hand, despite the absence of optimal methods to overcome this phenomenon of resistance, physicians must emphasize on proper patients compliance, as well as avoidance of potential drug-drug interactions. Finally one should always keep in mind that no single platelet activation pathway is responsible for all thrombotic complications, and a single treatment strategy directed against a specific receptor/target cannot overcome all thrombotic complications.

## Figures and Tables

**Table 1:** Prevalence of aspirin resistance.

Reference	Patients	Test used	Prevalence of AR	Comments
			*N*(%)	
Christiaens et al. [32]	*N* = 97	PFA-100 analyzer	29 (29.9)	♀ > ♂ (38 versus 15%)
	Stable CAD patients already on aspirin			No clinical correlation with laboratory parameters after 2.5 years follow-up

Pamukcu et al. [33]	*N* = 234	PFA-100 analyzer	52 (22.2)	Similar risk in resistant and nonresistant patients after 20.6 ± 6.9 months follow-up. Risk in aspirin resistant patients increased after cessation of clopidogrel
	Stable CAD			
			

Pamukcu et al. [34]	*N* = 105	PFA-100 analyzer	20 (19)	Greater risk of MACE in patients resistant to aspirin
	ACS			

Akay et al. [35]	*N* = 280	Optical platelet aggregometry	77 (27.5)	Large trial evaluating the frequency of AR in healthy subjects
	Healthy Turkish volunteers	(ADP, AA)		

Lee et al. [36]	*N* = 468	VerifyNow-Aspirin	128 (27.4)	100 mg or less daily dose were associated with a higher incidence of AR in patients with CAD
	Stable CAD			

Harrison et al. [30]	*N* = 100	PFA-100	22 (22)	Poor agreement between the different tests leads to the conclusion that aspirin resistance is highly test-specific
	Patients after TIA or Stroke	VerifyNow-Aspirin	17 (17)	
		Optical platelet aggregometry	5 (5)	

Gum et al. [28]	*N* = 325	Optical platelet aggregometry	18 (5.5)	Trend toward increased age in patients with AR
	Stable CAD	PFA-100 analyzer	31 (9.5)	

AR: aspirin resistance; CAD: coronary artery disease; ACS: acute coronary syndrome; MACE: major adverse cardicac events; ADP: adenosine diphosphate; AA: arachidonic acid; TIA: transient ischemic attack.

## References

[1] The Second International Study of Infarct Survival Collaborative Group (1988). Randomized trial of intravenous streptokinase, oral aspirin, both, or neither among 17,187 cases of suspected acute myocardial infarction: ISIS-2. *The Lancet*.

[2] Antithrombotic Trialists' Collaboration (1994). Collaborative overview of randomised trials of antiplatelet therapy—II: maintenance of vascular graft or arterial patency by antiplatelet therapy. *British Medical Journal*.

[3] Antithrombotic Trialists' Collaboration (1994). Collaborative overview of randomised trials of antiplatelet therapy—III: reduction in venous thrombosis and pulmonary embolism by antiplatelet prophylaxis among surgical and medical patients. *British Medical Journal*.

[4] Baigent C, Sudlow C, Collins R, Peto R (2002). Collaborative meta-analysis of randomised trials of antiplatelet therapy for prevention of death, myocardial infarction, and stroke in high risk patients. *British Medical Journal*.

[5] Marson P, Pasero G (2006). The Italian contributions to the history of salicylates. *Reumatismo*.

[6] Lévesque H, Lafont O (2000). Aspirin throughout the ages: an historical review. *La Revue de Médecine Interne*.

[7] Vane JR (2000). The fight against rheumatism: from willow bark to COX-1 sparing drugs. *Journal of Physiology and Pharmacology*.

[8] Vainio H, Morgan G (1997). Aspirin for the second hundred years: new uses for an old drug. *Pharmacology and Toxicology*.

[9] Patrono C, García Rodríguez LA, Landolfi R, Baigent C (2005). Low-dose aspirin for the prevention of atherothrombosis. *The New England Journal of Medicine*.

[10] Michelson AD, Cattaneo M, Eikelboom JW (2005). Aspirin resistance: position paper of the Working Group on Aspirin Resistance. *Journal of Thrombosis and Haemostasis*.

[11] Hankey GJ, Eikelboom JW (2006). Aspirin resistance. *The Lancet*.

[12] Wang TH, Bhatt DL, Topol EJ (2006). Aspirin and clopidogrel resistance: an emerging clinical entity. *European Heart Journal*.

[13] Michos ED, Ardehali R, Blumenthal RS, Lange RA, Ardehali H (2006). Aspirin and clopidogrel resistance. *Mayo Clinic Proceedings*.

[14] Michelson AD (2004). Platelet function testing in cardiovascular diseases. *Circulation*.

[15] Hennekens CH, Schror K, Weisman S, FitzGerald GA (2004). Terms and conditions: semantic complexity and aspirin resistance. *Circulation*.

[16] Mehta J, Mehta P, Burger C, Pepine CJ (1978). Platelet aggregation studies in coronary artery disease: past 4. Effect of aspirin. *Atherosclerosis*.

[17] Gurbel PA, Tantry US (2007). Clopidogrel resistance?. *Thrombosis Research*.

[18] André P, Delaney SM, LaRocca T (2003). P2Y_12_ regulates platelet adhesion/activation, thrombus growth, and thrombus stability in injured arteries. *The Journal of Clinical Investigation*.

[19] Sanderson S, Emery J, Baglin T, Kinmonth A-L (2005). Narrative review: aspirin resistance and its clinical implications. *Annals of Internal Medicine*.

[20] Macchi L, Sorel N, Christiaens L (2006). Aspirin resistance: definitions, mechanisms, prevalence, and clinical significance. *Current Pharmaceutical Design*.

[21] Shantsila E, Watson T, Lip GYH (2007). Aspirin resistance: what, why and when?. *Thrombosis Research*.

[22] Poulsen TS, Jørgensen B, Korsholm L, Licht PB, Haghfelt T, Mickley H (2007). Prevalence of aspirin resistance in patients with an evolving acute myocardial infarction. *Thrombosis Research*.

[23] Grau AJ, Reiners S, Lichy C, Buggle F, Ruf A (2003). Platelet function under aspirin, clopidogrel, and both after ischemic stroke: a case-crossover study. *Stroke*.

[24] Ziegler S, Maca T, Alt E, Speiser W, Schneider B, Minar E (2002). Monitoring of antiplatelet therapy with the PFA-100^®^ in peripheral angioplasty patients. *Platelets*.

[25] Grundmann K, Jaschonek K, Kleine B, Dichgans J, Topka H (2003). Aspirin non-responder status in patients with recurrent cerebral ischemic attacks. *Journal of Neurology*.

[26] Macchi L, Christiaens L, Brabant S (2002). Resistance to aspirin in vitro is associated with increased platelet sensitivity to adenosine diphosphate. *Thrombosis Research*.

[27] ten Berg JM, Gerritsen WBM, Haas FJLM, Kelder HC, Verheugt FWA, Thijs Plokker HW (2002). High-dose aspirin in addition to daily low-dose aspirin decreases platelet activation in patients before and after percutaneous coronary intervention. *Thrombosis Research*.

[28] Gum PA, Kottke-Marchant K, Poggio ED (2001). Profile and prevalence of aspirin resistance in patients with cardiovascular disease. *American Journal of Cardiology*.

[29] Andersen K, Hurlen M, Arnesen H, Seljeflot I (2002). Aspirin non-responsiveness as measured by PFA-100 in patients with coronary artery disease. *Thrombosis Research*.

[30] Harrison P, Segal H, Blasbery K, Furtado C, Silver L, Rothwell PM (2005). Screening for aspirin responsiveness after transient ischemic attack and stroke: comparison of 2 point-of-care platelet function tests with optical aggregometry. *Stroke*.

[31] Yee DL, Dinu BR, Sun CW (2008). Low prevalence and assay discordance of “aspirin resistance” in children. *Pediatric Blood & Cancer*.

[32] Christiaens L, Ragot S, Mergy J, Allal J, Macchi L (2008). Major clinical vascular events and aspirin-resistance status as determined by the PFA-100 method among patients with stable coronary artery disease: a prospective study. *Blood Coagulation & Fibrinolysis*.

[33] Pamukcu B, Oflaz H, Onur I (2007). Clinical relevance of aspirin resistance in patients with stable coronary artery disease: a prospective follow-up study (PROSPECTAR). *Blood Coagulation & Fibrinolysis*.

[34] Pamukcu B, Oflaz H, Oncul A (2006). The role of aspirin resistance on outcome in patients with acute coronary syndrome and the effect of clopidogrel therapy in the prevention of major cardiovascular events. *Journal of Thrombosis and Thrombolysis*.

[35] Akay OM, Canturk Z, Akin E, Bal C, Gulbas Z (2009). Aspirin-resistance frequency: a prospective study in 280 healthy Turkish volunteers. *Clinical and Applied Thrombosis/Hemostasis*.

[36] Lee P-Y, Chen W-H, Ng W (2005). Low-dose aspirin increases aspirin resistance in patients with coronary artery disease. *The American Journal of Medicine*.

[37] Rao GHR (1987). Influence of anti-platelet drugs on platelet-vessel wall interactions. *Prostaglandins, Leukotrienes and Medicine*.

[38] Koutts J (1990). Aspirin and coronary heart disease. Clinical applications. *Australian Family Physician*.

[39] Louden KA, Pipkin FB, Heptinstall S (1994). Neonatal platelet reactivity and serum thromboxane B2 production in whole blood: the effect of maternal low dose aspirin. *British Journal of Obstetrics and Gynaecology*.

[40] Bucchi F, Bodzento A, de Gaetano G, Cerletti C (1986). Effects of 1 gram oral or intravenous aspirin on urinary excretion of thromboxane B2 and 6-keto-PGF1*α* in healthy subjects. *Prostaglandins*.

[41] Frölich JC (1997). A classification of NSAIDs according to the relative inhibition of cyclooxygenase isoenzymes. *Trends in Pharmacological Sciences*.

[42] Cotter G, Shemesh E, Zehavi M (2004). Lack of aspirin effect: aspirin resistance or resistance to taking aspirin?. *American Heart Journal*.

[43] Biondi-Zoccai GGL, Lotrionte M, Agostoni P (2006). A systematic review and meta-analysis on the hazards of discontinuing or not adhering to aspirin among 50 279 patients at risk for coronary artery disease. *European Heart Journal*.

[44] Rottlaender D, Scherner M, Schneider T, Erdmann E (2007). Polypharmacy, compliance and non-prescription medication in patients with cardiovascular disease in Germany. *Deutsche Medizinische Wochenschrift*.

[45] Catella-Lawson F, Reilly MP, Kapoor SC (2001). Cyclooxygenase inhibitors and the antiplatelet effects of aspirin. *The New England Journal of Medicine*.

[46] Anand BS, Sanduja SK, Lichtenberger LM (1999). Effect of omeprazole on the bioavailability of aspirin: a randomized controlled study on healthy volunteers. *Gastroenterology*.

[47] Iñarrea P, Esteva F, Cornudella R, Lanas A (2000). Omeprazole does not interfere with the antiplatelet effect of low-dose aspirin in man. *Scandinavian Journal of Gastroenterology*.

[48] Halushka MK, Halushka PV (2002). Why are some individuals resistant to the cardioprotective effects of aspirin? Could it be thromboxane A_2_?. *Circulation*.

[49] Eikelboom JW, Hirsh J, Weitz JI, Johnston M, Yi Q, Yusuf S (2002). Aspirin-resistant thromboxane biosynthesis and the risk of myocardial infarction, stroke, or cardiovascular death in patients at high risk for cardiovascular events. *Circulation*.

[50] Goodman T, Ferro A, Sharma P (2008). Pharmacogenetics of aspirin resistance: a comprehensive systematic review. *British Journal of Clinical Pharmacology*.

[51] Szczeklik A, Undas A, Sanak M, Frolow M, Węgrzyn W (2000). Relationship between bleeding time, aspirin and the PlA1/A2 polymorphism of platelet glycoprotein IIIa. *British Journal of Haematology*.

[52] Papp E, Havasi V, Bene J (2005). Glycoprotein IIIA gene (*PlA*) polymorphism and aspirin resistance: is there any correlation?. *Annals of Pharmacotherapy*.

[53] Dropinski J, Musial J, Sanak M, Węgrzyn W, Nizankowski R, Szczeklik A (2007). Antithrombotic effects of aspirin based on PLA1/A2 glycoprotein IIIa polymorphism in patients with coronary artery disease. *Thrombosis Research*.

[54] Cohen HW, Crandall JP, Hailpern SM, Billett HH (2008). Aspirin resistance associated with HbA1c and obesity in diabetic patients. *Journal of Diabetes and Its Complications*.

[55] Ogawa H, Nakayama M, Morimoto T (2008). Low-dose aspirin for primary prevention of atherosclerotic events in patients with type 2 diabetes: a randomized controlled trial. *Journal of the American Medical Association*.

[56] Belch J, MacCuish A, Campbell I (2008). The prevention of progression of arterial disease and diabetes (POPADAD) trial: factorial randomised placebo controlled trial of aspirin and antioxidants in patients with diabetes and asymptomatic peripheral arterial disease. *British Medical Journal*.

[57] Zimmermann N, Wenk A, Kim U (2003). Functional and biochemical evaluation of platelet aspirin resistance after coronary artery bypass surgery. *Circulation*.

[58] Zimmermann N, Kurt M, Wenk A, Winter J, Gams E, Hohlfeld T (2005). Is cardiopulmonary bypass a reason for aspirin resistance after coronary artery bypass grafting?. *European Journal of Cardio-Thoracic Surgery*.

[59] Guthikonda S, Lev EI, Patel R (2007). Reticulated platelets and uninhibited COX-1 and COX-2 decrease the antiplatelet effects of aspirin. *Journal of Thrombosis and Haemostasis*.

[60] Modica A, Karlsson F, Mooe T (2007). Platelet aggregation and aspirin non-responsiveness increase when an acute coronary syndrome is complicated by an infection. *Journal of Thrombosis and Haemostasis*.

[61] Harrison P (2005). Platelet function analysis. *Blood Reviews*.

[62] Nicholson NS, Panzer-Knodle SG, Haas NF (1998). Assessment of platelet function assays. *American Heart Journal*.

[63] Gum PA, Kottke-Marchant K, Poggio ED (2001). Profile and prevalence of aspirin resistance in patients with cardiovascular disease. *American Journal of Cardiology*.

[64] Mammen EF, Comp PC, Gosselin R (1998). PFA-100^TM^ system: a new method for assessment of platelet dysfunction. *Seminars in Thrombosis and Hemostasis*.

[65] Christiaens L, Ragot S, Mergy J, Allal J, Macchi L (2008). Major clinical vascular events and aspirin-resistance status as determined by the PFA-100 method among patients with stable coronary artery disease: a prospective study. *Blood Coagulation & Fibrinolysis*.

[66] Coleman JL, Wang JC, Simon DI (2004). Determination of individual response to aspirin therapy using the Accumetrics Ultegra RFPA-ASA system. *Point of Care*.

[67] Nielsen HL, Kristensen SD, Thygesen SS (2008). Aspirin response evaluated by the VerifyNow^TM^ Aspirin System and Light Transmission Aggregometry. *Thrombosis Research*.

[68] Hart RG, Leonard AD, Talbert RL (2003). Aspirin dosage and thromboxane synthesis in patients with vascular disease. *Pharmacotherapy*.

[69] Bruno A, McConnell JP, Cohen SN (2004). Serial urinary 11-dehydrothromboxane B2, aspirin dose, and vascular events in 
blacks after recent cerebral infarction. *Stroke*.

[70] Lordkipanidzé M, Pharand C, Schampaert E, Turgeon J, Palisaitis DA, Diodati JG (2007). A comparison of six major platelet function tests to determine the prevalence of aspirin resistance in patients with stable coronary artery disease. *European Heart Journal*.

[71] Krasopoulos G, Brister SJ, Beattie WS, Buchanan MR (2008). Aspirin “resistance” and risk of cardiovascular morbidity: systematic review and meta-analysis. *British Medical Journal*.

[72] Snoep JD, Hovens MMC, Eikenboom JCJ, van der Bom JG, Huisman MV (2007). Association of laboratory-defined aspirin resistance with a higher risk of recurrent cardiovascular events: a systematic review and meta-analysis. *Archives of Internal Medicine*.

[73] Englyst NA, Horsfield G, Kwan J, Byrne CD (2008). Aspirin resistance is more common in lacunar strokes than embolic strokes and is related to stroke severity. *Journal of Cerebral Blood Flow and Metabolism*.

[74] Chen W-H, Cheng X, Lee P-Y (2007). Aspirin resistance and adverse clinical events in patients with coronary artery disease. *The American Journal of Medicine*.

[75] Chen W-H, Lee P-Y, Ng W, Tse H-F, Lau C-P (2004). Aspirin resistance is associated with a high incidence of myonecrosis after non-urgent percutaneous coronary intervention despite clopidogrel pretreatment. *Journal of the American College of Cardiology*.

[76] Frossard M, Fuchs I, Leitner JM (2004). Platelet function predicts myocardial damage in patients with acute myocardial infarction. *Circulation*.

[77] Campo G, Valgimigli M, Gemmati D (2006). Value of platelet reactivity in predicting response to treatment and clinical outcome in patients undergoing primary coronary intervention. Insights into the STRATEGY study. *Journal of the American College of Cardiology*.

[78] Barone-Rochette G, Ormezzano O, Polack B, Vanzetto G, Bertrand B, Machecourt J (2008). Resistance to platelet antiaggregants: an important cause of very late thrombosis of drug eluting stents? Observations from five cases. *Archives of Cardiovascular Diseases*.

[79] Faraday N, Yanek LR, Mathias R (2007). Heritability of platelet responsiveness to aspirin in activation pathways directly and indirectly related to cyclooxygenase-1. *Circulation*.

[80] Assadian A, Lax J, Meixner-Loicht U, Hagmüller GW, Bayer PM, Hübl W (2007). Aspirin resistance among long-term aspirin users after carotid endarterectomy and controls: flow cytometric measurement of aspirin-induced platelet inhibition. *Journal of Vascular Surgery*.

[81] Fitzgerald DJ, Maree A (2007). Aspirin and clopidogrel resistance. *Hematology*.

[82] Lee P-Y, Chen W-H, Ng W (2005). Low-dose aspirin increases aspirin resistance in patients with coronary artery disease. *The American Journal of Medicine*.

[83] Derry S, Loke YK (2000). Risk of gastrointestinal haemorrhage with long term use of aspirin: meta-analysis. *British Medical Journal*.

[84] Campbell CL, Smyth S, Montalescot G, Steinhubl SR (2007). Aspirin dose for the prevention of cardiovascular disease: a systematic review. *Journal of the American Medical Association*.

[85] Borna C, Lazarowski E, van Heusden C, Öhlin H, Erlinge D (2005). Resistance to aspirin in increased by ST-elevation myocardial infarction and correlates with adenosine diphosphate levels. *Thrombosis Journal*.

[86] Eikelboom JW, Hankey GJ, Thom J (2005). Enhanced antiplatelet effect of clopidogrel in patients whose platelets are least inhibited by aspirin: a randomized crossover trial. *Journal of Thrombosis and Haemostasis*.

[87] Wang TH, Bhatt DL, Fox KAA (2007). An analysis of mortality rates with dual-antiplatelet therapy in the primary prevention population of the CHARISMA trial. *European Heart Journal*.

[88] Bhatt DL, Flather MD, Hacke W (2007). Patients with prior myocardial infarction, stroke, or symptomatic peripheral arterial disease in the CHARISMA trial. *Journal of the American College of Cardiology*.

[89] The ESPS-2 Group (1997). European stroke prevention study 2: efficacy and safety data. *Journal of the Neurological Sciences*.

[90] Halkes PH, van Gijn J, Kappelle LJ, Koudstaal PJ, Algra A (2006). Aspirin plus dipyridamole versus aspirin alone after cerebral ischaemia of arterial origin (ESPRIT): randomised controlled trial. *The Lancet*.

[91] De Schryver E, Algra A, van Gijn J (2007). Dipyridamole for preventing stroke and other vascular events in patients with vascular disease. *Cochrane Database of Systematic Reviews*.

[92] Boncoraglio GB, Bodini A, Brambilla C, Corsini E, Carriero MR, Parati EA (2009). Aspirin resistance determined with PFA-100 does not predict new thrombotic events in patients with stable ischemic cerebrovascular disease. *Clinical Neurology and Neurosurgery*.

[93] Gent M (1996). A randomised, blinded, trial of clopidogrel versus aspirin in patients at risk of ischaemic events (CAPRIE). *The Lancet*.

[94] Sacco RL, Adams R, Albers G (2006). Guidelines for prevention of stroke in patients with ischemic stroke or transient ischemic attack: a statement for healthcare professionals from the American Heart Association/American Stroke Association council on stroke: co-sponsored by the council on cardiovascular radiology and intervention. The American Academy of Neurology affirms the value of this guideline. *Stroke*.

[95] Diener H-C, Bogousslavsky J, Brass LM (2004). Aspirin and clopidogrel compared with clopidogrel alone after recent ischaemic stroke or transient ischaemic attack in high-risk patients (MATCH): randomised, double-blind, placebo-controlled trial. *The Lancet*.

[96] Yusuf S, Zhao F, Mehta SR, Chrolavicius S, Tognoni G, Fox KK (2001). Effects of clopidogrel in addition to aspirin in patients with acute coronary syndromes without ST-segment elevation. *The New England Journal of Medicine*.

